# Human papillomavirus detection in women with and without human immunodeficiency virus infection in Colombia

**DOI:** 10.1186/1471-2407-14-451

**Published:** 2014-06-18

**Authors:** Milena Camargo, Sara C Soto-De Leon, Marina Munoz, Ricardo Sanchez, Diego Peña-Herrera, Andrea Clemencia Pineda-Peña, Otto Sussmann, Carol Paez, Antonio Perez-Prados, Manuel Elkin Patarroyo, Manuel Alfonso Patarroyo

**Affiliations:** 1 Fundación Instituto de Inmunología de Colombia, Cra. 50 # 26-20, Bogotá, Colombia; 2 School of Medicine and Health Sciences, Universidad del Rosario, Calle 14 # 6 – 25, Bogotá, Colombia; 3 Universidad Nacional de Colombia, Avenida Carrera 30 # 45, Bogotá, Colombia; 4 Northeastern University, 360 Huntington Ave, Boston, MA, USA; 5 Faculty of Natural and Mathematical Sciences, Universidad del Rosario, Calle 14 # 6 – 25, Bogotá, Colombia; 6 Asistencia Científica de Alta Complejidad S.A.S., Calle 45B # 24-25, Bogotá, Colombia; 7 Universidad Pública de Navarra, 31006 Pamplona, Spain

**Keywords:** Human papillomavirus, Human immunodeficiency virus, Multiple infection, Papanicolaou test, Epidemiology

## Abstract

**Background:**

HIV infection leads to a decreasing immune response, thereby facilitating the appearance of other infections, one of the most important ones being HPV. However, studies are needed for determining associations between immunodeficiency caused by HIV and/or the presence of HPV during the course of cervical lesions and their degree of malignancy. This study describes the cytological findings revealed by the Papanicolaou test, laboratory characteristics and HPV molecular profile in women with and without HIV infection.

**Methods:**

A total of 216 HIV-positive and 1,159 HIV-negative women were invited to participate in the study; PCR was used for the molecular detection of HPV in cervical samples. Statistical analysis (such as percentages, Chi-square test and Fisher’s exact test when applicable) determined human papillomavirus (HPV) infection frequency (single and multiple) and the distribution of six types of high-risk-HPV in women with and without HIV infection. Likewise, a logistic regression model was run to evaluate the relationship between HIV-HPV infection and different risk factors.

**Results:**

An association was found between the frequency of HPV infection and infection involving 2 or more HPV types (also known as multiple HPV infection) in HIV-positive women (69.0% and 54.2%, respectively); such frequency was greater than that found in HIV-negative women (44.3% and 22.7%, respectively). Statistically significant differences were observed between both groups (p = 0.001) regarding HPV presence (both in infection and multiple HPV infection). HPV-16 was the most prevalent type in the population being studied (p = 0.001); other viral types had variable distribution in both groups (HIV-positive and HIV-negative). HPV detection was associated with <500 cell/mm^3^ CD4-count (p = 0.004) and higher HIV-viral-load (p = 0.001). HPV-DNA detection, <200 cell/mm^3^ CD4-count (p = 0.001), and higher HIV-viral-load (p = 0.001) were associated with abnormal cytological findings.

**Conclusions:**

The HIV-1 positive population in this study had high multiple HPV infection prevalence. The results for this population group also suggested a greater association between HPV-DNA presence and cytological findings. HPV detection, together with low CD4 count, could represent useful tools for identifying HIV-positive women at risk of developing cervical lesions.

## Background

Epidemiological and molecular studies have led to a causal relationship being established between infection involving certain types of human papillomavirus (HPV), known as high risk (HR-HPV), and the development of cervical cancer (CC) [1]. Fifteen of the viral types identified to date have been classified as HR-HPV (HR-HPV-16, −18, −45, −31, −33, −52, −58 and −35 having the highest frequency) [1].

This type of cancer accounts for the second cause of death by malignant neoplasia in women, primarily those of childbearing age and living in developing countries. A CC mortality rate of 18.2 for every 100,000 women per year was reported in Colombia in 2010 [2].

HPV infection (considered a common sexually-transmitted infection) is necessary but not the sole factor for CC to develop; several risk factors can trigger the development of this cancer, immunosuppression being one of the most significant ones [3-5].

One of the most studied types of immunosuppression is that due to human immunodeficiency virus (HIV) infection [6]. Such infection leads to alterations in cell-mediated immunity, thereby facilitating the acquisition of opportunistic infections and limiting an organism’s ability to produce an efficient immune response [7].

Higher HPV infection prevalence has been documented in HIV-infected women; the foregoing, taken together with a deficient immune system, thus contributes towards new infections involving other HPV types being acquired (multiple infection or the coexistence of 2 or more HPV types) which, as the virus cannot be eliminated, increases HPV infection persistence in the body [5,8].

The clinical significance of HPV persistence concerns this virus’ long-term existence. This allows for the efficient replication and integration of viral oncoproteins (E6 and E7) in the host genome, mutations to become accumulated, genomic instability and chromosomal aberrations; this causes rapid morphological changes to cells, thereby contributing towards CC development [9,10].

Mild dysplasia prevalence in immunocompetent women does not exceed 27%, whereas this can rise to 80% in HIV-positive women; immunosuppression in the latter group implies the development of more aggressive lesions and a lower response to treatment [11], as well as leading to the appearance of precancerous lesions in about 60% of women suffering HPV infection which evolves in less than 3 years [11,12].

It has been described that HIV-positive women having a CD4 count of less than 200 cells/mm^3^ have greater HPV infection prevalence and increased cervical intraepithelial lesion incidence rates; the foregoing, together with a high HIV viral load, significantly increases the risk of developing this type of lesion [13].

It has been stated that the relationship between HIV-HPV might become a worldwide public health problem [14]; however, some reports have shown a low correlation between HIV infection and the development of neoplastic lesions. Such correlation mainly occurs in low-income countries having limited access to antiretroviral therapy (ART) [10,15].

No studies have been carried out to date in Colombia to determine HIV-HPV infection-related epidemiological profiles, so this study was aimed at describing the prevalence of multiple HPV infection, involving the type-specific distribution of six HR-HPV types amongst women with and without HIV infection involved in sexual and reproductive health programmes at four hospitals in Bogota. The resulting information has contributed towards knowledge regarding HIV-HPV-related sexually-transmitted infections.

## Methods

### Study design

This was a cross-sectional study; the patients participated between February 2007 and November 2012. All hospitals included in this study are located in the country’s capital city, Bogotá. The group of HIV-positive women (classified as having human immunodeficiency virus type 1) were attending a programme being run by Asistencia Científica de Alta Complejidad S.A.S (n = 216). The HIV-negative women were participating in sexual and reproductive health programmes being provided by state-run healthcare institutes; this group of women was recruited at the Hospital de Bosa (n = 232), Hospital de Fontibón (n = 290) and the Hospital de Engativá (n = 637), all located in Bogotá. The participating institutions reported the HIV status for all the women included in the study.

All the women were told about the study’s purpose. Those who volunteered to participate signed an informed consent form and completed a questionnaire aimed at collecting data concerning socio-demographic characteristics and risk factors before being given a gynaecological exam. An informed consent form, signed and approved in the presence of a parent or guardian, was required for women younger than 18 years old. This study was approved and supervised by all health care participating institutions’ Ethics Committees as follows: the Bioethics Committee of ‘Asistencia Científica de Alta Complejidad’, the Ethics Committee of the ‘Hospital de Bosa E.S.E.’, the Ethics Committee of the ‘Hospital de Fontibón E.S.E.’, and the Hospitalary Ethics Committee of the ‘Hospital de Engativá Nivel II’.

The inclusion criteria took into account the HIV status reported by the participating institutions, the women’s voluntary participation in this study by signing the consent form and filling in a questionnaire which considered socio-demographic characteristics and risk factors. Exclusion criteria considered women where a *β-globin* gene segment could not be amplified.

### Sample collection and methodological design

Papanicolaou test (Pap test) samples were collected following Colombian Ministry of Health guidelines for the early detection of CC [16]; the Pap smears were read by each participating hospital. Cytological findings were reported according to the Bethesda system as being atypical squamous cells of undetermined significance (ASCUS), a low grade squamous intraepithelial lesion (LSIL) or a high grade squamous intraepithelial lesion (HSIL).

Cervical samples for detecting HPV-DNA were obtained during cytological exam; cells were collected from the cervix using a cytobrush which was stored in a tube containing 95% ethanol as a means of preservation and transport [17].

The methodological design for detecting HPV involved using PCR, firstly, directed towards determining DNA quality and integrity (using primers directed towards detecting the *β-globin* gene segment). Independent PCRs were then used for the generic detection of HPV, using three sets of primers directed towards detecting segments encoding viral proteins L1 and E6-E7. Independent PCRs were also used on samples where generic detection had revealed the virus; type-specific identification involved using primers directed towards genes encoding early proteins E5, E6 and E7 [17,18].

### Sample processing and human papillomavirus DNA detection

Total DNA was extracted from cervical cells using a commercial QuickExtract solution kit (Epicentre, Madison, WI), following the manufacturer’s instructions.

PCR analysis involved GH20/PC04 and PC03/PC04 specific primers for detecting a *β-globin* gene segment in independent PCR reactions to confirm the presence of human DNA in all the samples [17].

Established PCR techniques were used for HPV-DNA detection [17,18]. Viral genes were detected using three primer sets; pU1M/2R was directed to the region encoding virus oncogenic proteins (E6/E7) and GP5+/6+ and MY09/11 directed towards a segment encoding the L1 late protein [17].

Samples having a positive result for HPV-DNA by PCR (samples proving positive for one or more of the three generic primer sets) were used for type-specific identification using primers targeting regions encoding viral oncogenic proteins E5, E6 or E7 from six high-risk HPV types (HR-HPV-16, −18, −31, −33, −45, −58), these being the most prevalent in the Colombian population and accounting for 90% of CC cases [17].

All PCRs were run simultaneously in separate reactions and in previously described conditions [17,18].

### Statistical analysis

Sample size was determined as being 179 in the HIV-positive group and 716 for the HIV-negative group to ensure 80% power for detecting a 1.60 odds ratio (OR). The proportion in group one (HIV-positive women) was assumed to be 0.45 by null hypothesis and 0.55 by alternative hypothesis.

A descriptive analysis of the demographic characteristics and risk factors was made and they were treated as categorical variables (mean, standard deviation (SD), percentages); estimations were made, along with their respective 95% confidence intervals (95% CI). Chi-square and Fisher’s exact tests were used (when applicable) to evaluate differences in proportions. The coexistence of 2 or more HPV types was defined as multiple infection in this study. The strength of association was measured using ORs with 95% CI; logistic regression was used for ordinal data to estimate adjusted ORs. The cofactors included in regression analysis were age, marital status, age at first intercourse, number of pregnancies, having had other STDs, the number of lifetime sexual partners, contraceptive methods used and smoking status, along with laboratory characteristics (Papanicolaou test, CD4-count, HIV-viral-load and ART). All hypothesis tests were set at 0.05 significance level. STATA11 was used for all statistical procedures.

## Results

The study involved 1,375 women aged 14–76 years (SD = 10.9; mean age = 36.9: 36.3-37.5 95% CI). The predominant ethnic group consisted of 1,337 mestizo women (97.2%: 96.2-98.1 95% CI), the remaining 38 (2.8%: 1.92-3.65 95% CI) being Afro-descendants and indigenous females.

216 of the women were HIV-positive whose ages ranged from 20–73 years old (SD = 10.7; mean age = 37.5: 36.0-38.9 95% CI) and a second group consisted of 1,159 HIV-negative women aged 14–76 (SD = 11.0; mean age = 36.8: 36.1-37.4 95% CI). The estimator revealed statistically significant differences regarding some women’s socio-demographic characteristics and risk factors according to HIV status (such as ethnicity and age at first intercourse), whilst no statistically significant difference was recorded for pregnancies (p = 0.173) and/or infection with other sexually transmitted diseases (p = 0.071) (Table 1).

**Table 1:** Demographic profile of the 1,375 women having positive human β-globin amplification

**Variable**	**HIV-positive**		**HIV-negative**		**p value**
	**(*n*** **= 216)**		**(*n*** **= 1,159)**		
Age*	37.5 [20–73]		36.8 [14–76]		
	SD = 10.7		SD = 11.0		
**Ethnicity**	***n***	**(%)**	***n***	**(%)**	
Indigenous	3	(1.4)	2	(0.2)	
Mestizo	209	(96.8)	1,128	(97.3)	
Afro-descendant	4	(1.8)	29	(2.5)	0.021
**Marital status**					
Single	66	(30.6)	301	(26.0)	
Married	36	(16.7)	202	(17.4)	
Cohabiting	65	(30.1)	497	(42.9)	
Separated	22	(10.2)	132	(11.4)	
Widowed	27	(12.4)	27	(2.3)	0.001
**Age at first intercourse**					
<15	75	(34.7)	276	(23.8)	
16-19	111	(51.4)	618	(53.3)	
>19	30	(13.9)	265	(22.9)	0.001
**Pregnancies**					
None	10	(4.6)	69	(6.0)	
1-3	160	(74.1)	784	(67.6)	
>4	46	(21.3)	306	(26.4)	0.173
**Abortions**					
No	120	(55.7)	741	(64.0)	
Yes	96	(44.3)	418	(36.0)	0.019
**Other STD**					
No	136	(63.0)	653	(56.4)	
Yes	80	(37.0)	506	(43.6)	0.071
**Lifetime number of sexual partners**					
1-2	88	(40.7)	783	(67.5)	
>3	128	(59.3)	376	(32.5)	0.001
**Contraceptive method**					
None	43	(19.9)	337	(29.1)	
Hormonal	11	(5.1)	189	(16.3)	
Surgery	46	(21.3)	318	(27.4)	
Condom	97	(45.0)	113	(9.8)	
Intrauterine device	19	(8.7)	202	(17.4)	0.001
**Smoking status**					
No	177	(81.9)	776	(66.9)	
Yes	39	(18.1)	383	(33.1)	0.001
**Cytological findings**					
Normal	148	(68.5)	918	(79.2)	
Abnormal	68	(31.5)	241	(20.8)	0.006

p value: Probability value, SD: standard deviation, STD: sexually transmitted diseases.

*Mean [range]; SD.

All samples (n = 1,375) which amplified for the *β-globin* gene and tested for HPV-DNA presence (indicated by positive amplification of pU1M/2R, GP5+/6+ or MY09/11, or more than one) were used in the statistical analysis. The virus was detected in 48.1% of the samples (n = 662: 45.5-50.8 95% CI) and multiple infection (defined as coexistence of 2 or more HPV types) in 57.4% (n = 380: 53.5-61.7 95% CI) of HPV positive samples.

HPV-DNA was detected in 44.3% of samples from HIV-negative women (n = 513: 41.4-47.2 95% CI) and multiple infection in 51.3% of them (n = 263: 46.8-55.7 95% CI). The presence of HPV was observed in 69.0% of HIV-positive women (n = 149: 62.3-75.0 95% CI) and multiple infection was found in 78.5% of the sample (n = 117: 71.0-84.8 95% CI). Statistically significant differences were observed between both groups of women (p = 0.001) regarding HPV presence (both in infection and multiple HPV infection).

Cytological findings proved negative for intraepithelial lesions or malignancy in 68.5% of the HIV-positive women (n = 148: 61.9-74.7 95% CI) and abnormality occurred in 31.5% of them (n = 68: 25.3-38.1 95% CI), classified as follows: ASCUS occurred in 35.3% of cytological abnormalities (n = 24: 24.1-47.8 95% CI), LSIL in 57.3% (n = 39: 44.8-69.3 95% CI) and HSIL in 7.4% of them (n = 5: 2.4-16.3 95% CI).

79.2% (n = 918: 76.7-81.5 95% CI) of HIV-negative women proved negative for lesions, whilst 20.8% of them (n = 241: 18.5-23.2 95% CI) had some degree of abnormality: 52.7% (n = 127: 46.2-59.1 95% CI) had ASCUS, 41.5% (n = 100: 35.2-47.9 95% CI) had LSIL and 5.8% (n = 14: 3.21-9.55 95% CI) HSIL. The difference between abnormality detected by Pap test for women having different HIV infection status was statistically significant (p = 0.003).

Association between HPV status and Pap test result determined by ORs revealed a positive trend for HPV-DNA detection and ASCUS cytological findings (crude OR 1.85: 1.31-2.63 95% CI). A positive association was observed when comparing SIL cytological findings (crude OR 1.70: 1.21-2.39 95% CI; test of trends in odds: Chi^2^ (1) = 16.13 p = 0.001). Calculating regression analysis association between HIV status and Pap test result revealed a positive trend for HIV-positive and SIL (crude OR 2.39: 1.61-3.54 95% CI; test of trends in odds: Chi^2^ (1) = 18.04 p = 0.001). All associations remained statistically significant after using logistical regression to adjust for cofactors (described in the statistical analysis section) (Table 2).

**Table 2:** Multivariate analysis of HPV status and HIV status in women regarding cytological findings

	**HPV status**						**HIV status**					
	**Negative**		**Positive**		**Adjusted OR (95% CI)**		**Negative**		**Positive**		**Adjusted OR (95% CI)**	
**Pap test result**	**n**	**(%)**	**n**	**(%)**			**n**	**(%)**	**n**	**(%)**		
**Negative**	587	(82.3)	479	(72.4)	Ref		918	(79.2)	148	(68.5)	Ref	
**ASCUS**	60	(8.4)	91	(13.7)	**1.84**	**1.28-2.62**	127	(11.0)	24	(11.1)	1.18	0.70-1.99
SIL*	66	(9.3)	92	(13.9)	**1.69**	**1.19-2.41**	114	(9.8)	44	(20.4)	**2.74**	**1.74-4.33**
**Total**	713		662				1,159		216			

OR adjusted for age, marital status, age at first intercourse, the number of lifetime sexual partners, contraceptive methods used and smoking status, Papanicolaou test, CD4-count, HIV-viral-load and antiretroviral therapy or ART.

95% CI: confidence interval, OR: odds ratio, ASCUS: atypical squamous cells of undetermined significance, SIL: squamous intraepithelial lesions.

Values in bold = p < 0.05.

*SIL include: low grade squamous intraepithelial lesion (LSIL) and high grade squamous intraepithelial lesions (HSIL).

Positive associations (using regression analysis) between abnormal cytological findings (including ASCUS, LSIL and HSIL) and clinical and laboratory characteristics (CD4 count, HIV viral load and antiretroviral therapy-ART) showed a positive trend for CD4 cell count below 200 cell/mm^3^ (crude OR 2.96: 1.85-4.73 95% CI; test of trends in odds: Chi^2^ (1) = 23.03 p = 0.001), higher than 100,000 copies/mL HIV viral load (crude OR 10.47: 3.26-33.60 95% CI; test of trends in odds: Chi^2^ (1) = 23.40 p = 0.001) and women with and without ART treatment (crude OR 1.57: 1.11-2.21 95% CI, and crude OR 4.19: 1.75-10.03 95% CI; test of trends in odds: Chi^2^ (1) = 15.96 p = 0.001) (Table 3).

**Table 3:** Multivariate analysis of factors associated with abnormal cytological findings

	**Abnormal Pap test result** *			
	**n**	(%)**	**Adjusted OR**	**95% CI**
**HIV; immune status CD4 cell/mm**^**3 **^**count**				
HIV-negative	241	(20.8)	Ref	
HIV-positive, < 200	35	(16.2)	**2.89**	**1.73-4.84**
HIV-positive, 200 - 349	12	(5.6)	1.05	0.52-2.11
HIV-positive, 350 - 500	12	(5.6)	1.34	0.66-2.71
HIV-positive, > 500	9	(4.2)	1.41	0.63-3.16
**HIV viral load copies/mL**				
HIV-negative	241	(20.8)	Ref	
HIV-positive, <4,000	49	(22.7)	1.44	0.96-2.16
HIV-positive, 4,000-99,999	8	(3.7)	1.95	0.77-4.94
HIV-positive, >100,000	11	(5.1)	**8.63**	**2.65-28.12**
**HIV and ART use**				
HIV-negative	241	(20.8)	Ref	
HIV-positive, treatment with ART	57	(26.4)	**1.57**	**1.06-2.32**
HIV-positive, without treatment with ART	11	(5.1)	**3.63**	**1.39-9.45**

OR adjusted for age, marital status, age at first intercourse, the number of lifetime sexual partners, contraceptive methods used and smoking status, Papanicolaou test, CD4-count, HIV-viral-load and antiretroviral therapy or ART.

Values in bold = p < 0.05.

Pap test: Papanicolaou test, 95% CI: confidence interval, OR: odds ratio, HIV: Human Immunodeficiency Virus, ART: antiretroviral therapy, ASCUS: atypical squamous cells of undetermined significance, LSIL: low grade squamous intraepithelial lesion, HSIL: high grade squamous intraepithelial lesions.

*Includes ASCUS, LSIL and HSIL.

**Percentages were calculated regarding HIV-positive = 216 and HIV-negative n = 1,159.

ORs were used for assessing the association between women with and without HIV infection and having HPV (prevalence, infection status, clade detected and clinical and laboratory characteristics). The results revealed statistically significant associations between HIV-positive women and an increased likelihood of HPV-DNA detection (crude OR 2.80: 2.04-3.84 95% CI; p = 0.001), multiple infection (crude OR 4.02: 2.94-5.49 95% CI; p = 0.001), clade 7 (crude OR 2.62: 1.90-3.62 95% CI; p = 0.001) and clade 9 detection (crude OR 2.91: 2.14-3.97 95% CI; p = 0.001), lower than 500 cell/mm^3^ CD4 cell count (crude OR 2.02: 1.11-3.70 95% CI; p = 0.004), higher than 100,000 copies/mL HIV viral load (crude OR 8.18: 1.82-36.71 95%C; p = 0.001) and women with and without ART treatment (crude OR 2.68: 1.93-3.72 95% CI; p = 0.001 and crude OR 3.52: 1.25-9.88 95% CI; p = 0.005). These associations remained significant in multivariate analysis (Table 4).

**Table 4:** Relative frequency and multivariate analysis of HPV infection in HIV- negative and HIV- positive women

	**HPV positive detection**			
	**n**	(%)*	**Adjusted OR**	**95% CI**
**HIV status**				
Negative	513	(44.3)	Ref	
Positive	149	(69.0)	**2.37**	**1.69-3.34**
**HIV and one type only HPV (single infection)**				
HIV-negative, single infection	250	(21.6)	Ref	
HIV-positive, single infection	32	(14.8)	1.14	0.85-1.28
**HIV and >1 type HPV (multiple infection)**				
HIV-negative, multiple infection	263	(22.7)	Ref	
HIV-positive, multiple infection	117	(54.2)	**3.43**	**2.39-4.92**
**HIV and HPV Clade 7**				
HIV-negative, HPV Clade 7	202	(17.4)	Ref	
HIV-positive, HPV Clade 7	77	(35.7)	**2.48**	**1.69-3.64**
**HIV and HPV Clade 9**				
HIV-negative, HPV Clade 9	454	(39.2)	Ref	
HIV-positive, HPV Clade 9	141	(65.3)	**2.47**	**1.73-3.53**
**HIV; immune status CD4 cell/mm**^**3 **^**count**				
HIV-negative	513	(44.3)	Ref	
HIV-positive, < 200	63	(29.2)	**4.38**	**2.47-7.77**
HIV-positive, 200 - 349	39	(18.1)	**3.13**	**1.64-5.96**
HIV-positive, 350 - 500	29	(13.4)	**1.91**	**1.03-3.57**
HIV-positive, > 500	18	(8.3)	1.17	0.59-2.32
**HIV viral load copies/mL**				
HIV-negative	513	(44.3)	Ref	
HIV-positive, <4,000	121	(56.0)	2.41	0.67-3.48
HIV-positive, 4,000-99,999	15	(6.9)	2.43	0.96-6.14
HIV-positive, >100,000	13	(6.0)	**7.32**	**1.62-33.03**
**HIV and ART use**				
HIV-negative	513	(44.3)	Ref	
HIV-positive, treatment with ART	133	(61.6)	**2.46**	**1.72-3.51**
HIV-positive , without treatment with ART	16	(7.4)	**3.32**	**1.16-9.50**

OR adjusted for age, marital status, age at first intercourse, the number of lifetime sexual partners, contraceptive methods used and smoking status, Papanicolaou test, CD4-count, HIV-viral-load and antiretroviral therapy or ART. Abbreviations: 95% CI: confidence interval, OR: odds ratio, HIV: Human Immunodeficiency Virus, ART: antiretroviral therapy, HPV: Human Papillomavirus.

*Percentages were calculated regarding HIV-positive = 216 and HIV-negative n = 1,159.

Clade 7: HPV-18 and −45; Clade 9: HPV-16, −31, −33 and −58. Values in bold = p < 0.05.

Regarding type-specific distribution in HIV-positive and negative groups, HPV-16 was the most prevalent infection for the entire study population (p = 0.001). Significant differences were observed regarding the percentages of HPV infection types for each population, HPV-31 was the second most prevalent in HIV-positive women (p = 0.001), HPV-18 the third (p = 0.001) (Table 5) whilst HPV-58 was the second most prevalent in HIV-negative women (p = 0.215) followed by HPV-31 (p = 0.001). HPV-45 occurred least frequently in both populations (p = 0.001) (Table 5).

**Table 5:** Relative frequency of HPV infection and type-specific distribution in HIV-positive and HIV-negative women

	**HPV positive**		**HPV-type**											
			**HPV-16**		**HPV-18**		**HPV-31**		**HPV-33**		**HPV-45**		**HPV-58**	
**HIV-positive (n = 216)**	**n**	**(%)**	**n**	**(%)**	**n**	**(%)**	**n**	**(%)**	**n**	**(%)**	**n**	**(%)**	**n**	**(%)**
	149	69.0	100	46.3	66	30.6	71	32.9	40	18.6	19	8.8	44	20.4
**CD4 cell/mm**^**3 **^**count**														
< 200	63	29.2	44	20.4	22	10.2	35	16.2	13	6.0	9	4.2	18	8.3
200 - 349	39	18.1	22	10.2	18	8.3	19	8.8	15	6.9	5	2.3	14	6.5
350 - 500	29	13.4	18	8.3	20	9.3	12	5.6	10	4.6	3	1.4	8	3.7
> 500	18	8.3	16	7.4	6	2.8	5	2.3	2	0.9	2	0.9	4	1.9
**HIV viral load copies/mL**														
<4,000	121	56.0	82	38.0	54	25.0	54	25.0	33	15.3	15	6.9	32	14.8
4,000-99,999	15	6.9	11	5.1	9	4.2	10	4.6	4	1.9	1	0.5	8	3.7
>100,000	13	6.0	7	3.2	3	1.4	7	3.2	3	1.4	3	1.4	4	1.9
**ART use**														
Yes	133	61.6	90	41.7	61	28.2	64	29.6	36	16.7	18	8.3	39	18.1
No	16	7.4	10	4.6	5	2.3	7	3.2	4	1.9	1	0.5	5	2.3
**HIV-negative (n = 1,159)**														
	513	44.3	222	19.2	118	10.2	151	13.0	104	9.0	98	8.5	177	15.3

HIV: Human Immunodeficiency Virus, ART: antiretroviral therapy, HPV: Human Papillomavirus.

Significant differences were observed concerning HPV type distribution regarding 200 to 349 cell/mm^3^ CD4 count; HPV-18 had the greatest prevalence (p = 0.041), followed by HPV-16 (p = 0.241) and HPV-31 (p = 0.008). Other differential distributions were observed for higher than 100,000 copies/mL HIV viral load; however, they were not statistically significant (Table 5).

## Discussion

HPV infection and multiple infection prevalence was found to be similar to that reported for Colombia in previous studies concerned with the heterogeneous female population [17].

HPV prevalence in the HIV-positive population was significantly higher than in the group of HIV-negative women. This agreed with other studies which found that HIV-related immunosuppression disabled an immune system response to concomitant infection [4,19].

High HPV infection prevalence in HIV-positive women has been reported in worldwide studies; there is 44% HPV infection in European countries such as Italy [5] and US studies have found 54%-73% prevalence [20,21], similar to that for Latin-American countries like Brazil where 48% to 68% HPV prevalence has been found in HIV-positive women [22,23].

Our results showed that multiple HPV infection occurred more frequently in HIV-positive women; this may have been due to a deficient immune system [7], risky sexual behaviour engaged in by this particular group involving an increased exposure risk and higher reactivation rates regarding latent HPV infection [7,19], Furthermore, it has been reported that the risk of acquiring later HPV infection becomes increased in HPV-infected women, which could contribute to high multiple infection frequency [24].

Some studies have shown the co-existence of more than one HPV type in the same organism [25]; however, the clinical relevance of being infected with multiple HPV types has not been clearly established. Multiple infection results have indicated that HPV facilitates persistence at the site of infection, leading to an increased risk of premalignant lesions progressing to CC [24,26].

Type-specific distribution revealed that HPV-16 was the most prevalent type in the population being studied; this was consistent with worldwide results to date [17,27,28]. High prevalence was found for the other HPV types being tested, such as HPV-31 in HIV-positive women and HPV-58 in the group of HIV-negative females; these viral types have been reported in Colombia in previous studies by our research group and others working in the field as being the most prevalent [17,29].

Such differential type distribution between groups (HIV-positive and HIV-negative) may have been partly due to immune system deficiency, thereby contributing towards the inefficient removal of the virus or evasion by certain types of HPV. The latter benefits the colonisation and persistence of some viral types (mainly oncogenic HPV) [7,8,30].

A positive association was seen in the current study for women having lower than 200 cell/mm^3^ CD4 count with abnormal cytological findings, compared to those having higher CD4 count, thereby agreeing with a previous report [5]. Lower than 200 cell/mm^3^ count indicates a deficient immune system and, together with HPV detection, could be used as predictors of pre-neoplastic cervical lesions [31].

Our groups’ results showed that a lower than 500 cell/mm^3^ CD4 count was associated with HPV detection, thereby reflecting the inability of HIV-positive women’s immune systems to respond to opportunist infection. High HIV viral load and low CD4 cell counts could facilitate acquiring HPV infection [32,33].

The effect of antiretroviral drugs on the incidence of cervical lesions having a poor prognosis has not been clearly established; previous studies, such as the US WIHS (Women’s Interagency HIV Study) cohort, have indicated that ART treatment for HIV positive women has led to duration and progression becoming reduced [31]. Our group’s results showed that women with and without treatment with antiretroviral drugs had a significant association with HPV detection and abnormal cytological findings; this could have been due to high HPV-16 prevalence, since it has been established that the effect of therapy involving ART on the appearance of cervical disease is reduced when infections by this viral type occur [31].

More lesions were detected in the HIV-positive group (mainly LSIL); studies have indicated that women having this type of immunosuppression have a higher incidence and prevalence of premalignant lesions caused by the immune system becoming unable to efficiently eliminate HPV infection. This also favours the virus’ persistence in the body, ultimately contributing towards the development of this type of dysplasia [34,35].

Association studies have shown that HIV infection increases the risk of CC development (up to 22-fold) compared to such risk in the general population [19]. The natural history of CC development becomes altered in women having immunosuppression caused by HIV since the regression of lesions has been seen to decrease significantly compared to HIV-negative women [4,36].

This can be explained as immunosuppression leads to an alteration in local and systemic immune response, thereby preventing suitable clearance of HPV infection. This results in recurrent pre-neoplastic lesions and lower regression rates; however, it has not been clearly established whether this is the main mechanism [6,13,21].

Our results showed a significant association between infection involving clade 9 and HIV-positive women; this may have been partly due to HPV-16 in this clade being the most prevalent viral type in such population. This viral type has been shown to have an association with the risk of acquiring other HPV types; however, it has not been clearly established whether a higher risk of acquiring a phylogenetically-related type is associated with the risk of acquiring another type of HPV [7,24].

Interestingly, a positive trend was found between ASCUS cytology and HPV-DNA being detected. ASCUS cytology indicated that cytological-morphological changes were not benign; however, this did not meet the criteria for classification as squamous lesions, even though studies have shown that this type of cytological interpretation is highly related to characteristic subjective reading of Pap tests [37,38].

Studies have found that HPV detection in women having ASCUS has provided high sensitivity in detecting severe dysplasia and cancer [39]. This is why strengthening HPV molecular screening for detecting women having atypical ASCUS (first indicator of cervical abnormalities) may help in reducing the impact caused by CC on an immunocompetent population, as well as HIV-infected women who are at increased risk [40].

One of this study’s limitations lay in the detection of 6 of the 15 HPV types described as being high risk. Even though the types identified in the present study are considered to be prevalent in the Colombian population, other types of high and low risk HPV should be included in future studies. This study’s cross-sectional design did not lead to determining a causal relationship between the immunodeficiency caused by HIV and/or the prevalence and persistence of HPV.

## Conclusions

There is limited information in Colombia regarding HPV infection in HIV-positive women. This is the first study in Colombia which has evaluated epidemiological profiles concerning HPV infection in both HIV-positive and HIV-negative women. The results further showed multiple HPV infection as an associated factor in HIV-positive women; however, further prospective studies are needed to determine the dynamics and follow-up patterns (i.e. 5-year follow-up) for these infections and their influence on the development of cervical dysplasia.

HR-HPV distribution in women should be studied to facilitate developing prevention and management strategies in the general population, prioritising HIV-positive women as they represent a special group due to their type of immunosuppression altering the natural course of CC development. The resulting information has contributed towards knowledge regarding HIV-HPV-related sexually-transmitted infections.

## Abbreviations

HPV: Human papillomavirus; HIV: Human immunodeficiency virus; ART: Antiretroviral therapy; HR-HPV: High risk human papillomavirus; LR-HPV: Low risk human papillomavirus; DNA: Deoxyribonucleic acid; CC: Cervical cancer; SIL: Squamous intraepithelial lesions; ASCUS: Atypical squamous cells of undetermined significance; LSIL: Low grade squamous intraepithelial lesion; HSIL: High grade squamous intraepithelial lesion; STD: Sexually-transmitted diseases; Pap test: Papanicolaou test; 95% CI: Confidence interval; OR: Odds ratio; SD: Standard deviation; PCR: Polymerase chain reaction.

## Competing interests

The authors declare that they have no competing interests.

## Authors’ contributions

MC was involved in the design of the study, helped in the collection of clinical data, performed the molecular tests and analysis and drafted the manuscript. SCSL was involved in the design of the study, helped in the collection of clinical data, performed the molecular tests and analysis and drafted the manuscript. MM was involved in the design of the study, helped in the collection of clinical data, performed the molecular tests and analysis and drafted the manuscript. RS conducted statistical analyses and contributed to the manuscript. DPH was involved in the analysis and interpretation of data. ACPP collected the clinical data and made a critical review of the manuscript. OS collected the clinical data and made a critical review of the manuscript. CP collected the clinical data and made a critical review of the manuscript. APP was involved in the analysis and interpretation of data. MEP co-designed the study and MAP co-designed the study, led the research project and proofread the final document. All authors read and approved the final manuscript.

## Pre-publication history

The pre-publication history for this paper can be accessed here:

http://www.biomedcentral.com/1471-2407/14/451/prepub
